# Can probiotic, prebiotic, and synbiotic supplementation modulate the gut-liver axis in type 2 diabetes? A narrative and systematic review of clinical trials

**DOI:** 10.3389/fnut.2022.1052619

**Published:** 2022-12-01

**Authors:** Yousef Al-Najjar, Maryam Arabi, Pradipta Paul, Ali Chaari

**Affiliations:** ^1^Medical Education Division, Weill Cornell Medicine-Qatar, Cornell University, Qatar Foundation – Education City, Doha, Qatar; ^2^Premedical Division, Weill Cornell Medicine-Qatar, Qatar Foundation – Education City, Doha, Qatar

**Keywords:** gut microbiome, dysbiosis, nutraceutical, clinical trial, liver function

## Abstract

**Background:**

Type 2 diabetes, one of the most common noncommunicable diseases, is a metabolic disorder that results in failed homeostatic control in several body systems, including hepatic function. Due to the gut microbiome’s potential role in diabetes’ pathogenesis, prebiotics, probiotics, and synbiotics have been proposed as complimentary therapeutic approaches aimed at microbiota readjustment.

**Methods:**

A systematic review was conducted on PubMed, Scopus, Web of Science, Embase, and the Cochrane Library examining the effect of probiotics, prebiotics, and synbiotics on hepatic biomarkers in patients with diabetes.

**Results:**

From 9,502 search hits, 10 studies met the inclusion criteria and were included in this review. A total of 816 participants (460 intervention and 356 control) were investigated for the effects of nine different hepatic biomarker measurements including aspartate aminotransferase, alanine aminotransferase, alkaline phosphatase, total protein, bilirubin, liver steatosis, liver stiffness, fatty liver index, and gamma-glutamyl transferase levels. Of the 13 intervention groups analyzed from the 10 studies, 3 were prebiotic interventions, 3 were single species probiotic interventions, 3 were multi-species probiotic interventions, and 4 were synbiotic interventions. Nutraceuticals used in these trials included six genera of bacteria (*Lactobacillus, Bifidobacterium, Streptococcus, Acetobacter, Lactococcus*, and *Propionibacterium*), five different prebiotic formulations (inulin, inulin and beta carotene, chicory inulin enriched with oligofructose, galacto-oligosaccharides syrup, and powdered cinnamon), or a combination of these to form multi-species probiotics or synbiotics.

**Conclusion:**

Although some studies showed insignificant changes in hepatic biomarkers, generally the results yielded a decrease in liver damage due to reduced oxidative stress, pro-inflammatory cytokines, gut dysbiosis, and insulin resistance which led to improvements in hepatic biomarker levels.

## Introduction

Type 2 diabetes mellitus (T2DM) is an ever-growing global health concern. In 2021, its prevalence globally in 20–79-year-old individuals was approximated to be 536.6 million individuals, representing 10.5% of global population (1). These figures are expected to rise to 783.2 million cases (12.2%) by 2045 (1). Genetic predispositions, environmental influences, metabolic disorders, and aging are strongly associated with the onset of T2DM and are therefore intertwined with these rising trends (2). In 2019, T2DM was found to be the root cause of 1.5 million deaths worldwide, with 48% of these fatal outcomes occurring in patients under the age of 70 (3). In addition to considerable mortality and morbidity, T2DM also takes a heavy financial toll on healthcare systems: the total expenditure of diagnosed T2DM was estimated to be around 327 billion USD in 2017, signifying a 26% increase over a period of 5 years (4).

The American Diabetes Association (ADA) and the European Association for the Study of Diabetes (EASD) emphasize the need for both lifestyle changes and pharmacological drugs in the long-term control of T2DM (5). Nonetheless, drugs that are currently used over a long period of time have many drawbacks, especially those that are used for insulin replacement (6). These drawbacks include the inability to inhibit the autoimmune response that causes the impairment of pancreatic β-cells, and ineffectiveness in preventing various diabetic complications, including cardiovascular problems (7). Most antidiabetic drugs aim to stabilize blood glucose levels in patients with T2DM, but probiotics, prebiotics, and synbiotics could be used as complimentary or adjunct therapies *via* dietary intervention to treat one of the root causes – gut dysbiosis. Several studies were conducted on the effects of these supplements on the gut microbiota in diabetic patients and revealed significant improvements in patients’ inflammatory and oxidative, glycemic, and lipid profiles (8–10). These improvements are due to the supplements’ ability to counter bacterial translocation, reduce chronic inflammation, and enhance the body’s metabolic status (11).

## Disruption of the gut-liver axis in diabetes

The indigenous bacterial population of the human intestine is composed of around 100 trillion bacteria, almost 10 times more cells than there are in the human body (12). Of these bacteria, 35,000 species were identified and categorized into six phyla: Firmicutes, Bacteroidetes, Actinobacteria, Proteobacteria, Fusobacteria, and Verrucomicrobia (13). One of the primary functions of the gut microbiota is to increase the energy available from organic polymers otherwise resistant to digestion by human enzymes. For instance, the phylum Bacteroidetes is believed to aid in the digestion of plant cell wall compounds, N-glycans and O-glycans, thereby unlocking energy *via* the release of short chain fatty acids (SCFAs), which account for 7–10% of daily caloric requirements (14, 15). SCFAs, such as butyrate, acetate, and propionate, are not only largely used as a source of energy, but are also crucial modulators of several physiological pathways, including the adaptive immunity’s anti-inflammatory response *via* the downregulation of inflammatory cytokines such as tumor necrosis factor-alpha (TNF-α) and IL-1β (16). The metabolites made by SCFA-producing bacteria in the gut have also been implicated in the maintenance of intestinal integrity and stimulating mucus production (17).

**FIGURE 1:**
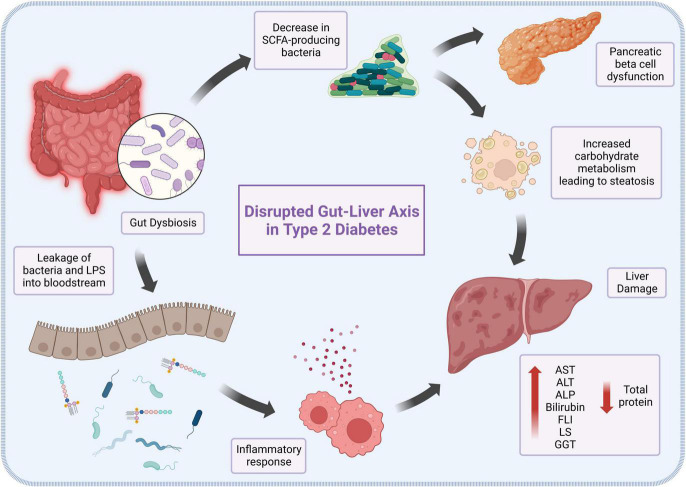
The role of gut dysbiosis in the pathogenesis and progression of type 2 diabetes-induced liver damage.

This collectively makes the gastrointestinal tract an important component in the structural, humoral, and physiological development of the body, as well as in the progression of diseases such as T2DM, as shown in Figure 1. Several studies have demonstrated the association between T2DM and gut microbial dysbiosis, such as an observed decrease in the amount of SCFA-producing bacteria in conjunction with a rise in opportunistic bacteria in patients living with T2DM (18). The effects of a lack of SCFA in T2DM patients are complex and varied, ranging from impairment of receptor-mediated signaling in pancreatic beta cells, to dysregulation of the gut-brain axis and hepatic encephalopathy (19). Furthermore, it has been suggested that the translocation and ratio alterations of the intestinal microbiome in diabetic and obese patients may lead to the metabolism of otherwise insoluble and indigestible carbohydrates, significantly raising energy harvesting and adiposity in the liver (20). Dysbiosis is also involved in chronic inflammation and oxidative stress (21). This is partly due to the production and release of endotoxins, including lipopolysaccharides (LPS), as well as the translocation of intestinal bacteria from the gut microbiota into the bloodstream (22). These bacteria and bacteria-derived LPS travel through the bloodstream, where LPS binds to Toll-like receptor 4 (TLR4) on all hepatocytes, especially Kupffer cells (23). This interaction causes the release of TNF-α, which induces the hepatic inflammatory responses and liver fibrosis associated with T2DM, and the progression from non-alcoholic fatty liver disease to nonalcoholic steatohepatitis (NASH) (24). As the liver becomes more severely damaged, enzymes such as transaminases, which are usually located inside the hepatocytes, are released into the serum from the impaired cells, increasing their levels in serum (25). On the other hand, proteins such as albumin that are normally manufactured and secreted by the liver are no longer produced due to destruction of cell machinery, leading to their decreased serum levels (26).

## The various hepatic biomarkers

Medical tests used to evaluate liver function include serum biomarkers of injury [e.g., transaminases, γ-glutamyl transferase, and alkaline phosphatase (ALP)], hepatic dysfunction [e.g., liver stiffness (LS)], and antioxidants (e.g., bilirubin). Such biomarkers are utilized as proxies for liver health, one of the affected physiologies in T2DM (27).

### Transaminases

Aminotransferases, or transaminases, are enzymes that catalyze the transfer of amino groups in the reversible conversion of amino acids and oxoacids (28). Among many transaminases, alanine transaminase (ALT) and aspartate transaminase (AST) are two enzymes that are clinically significant, especially in liver function tests (29). Damage to hepatocytes causes high activity levels of serum aminotransferases in T2DM and is therefore used as a diagnostic tool for insulin resistance and metabolic syndrome (30). This is due to disruption of the cell plasma membranes of liver cells, causing leakage of these enzymes (31).

### Alkaline phosphatase

Aside from aminotransferases, ALP is also considered to be an independent risk factor for the development of T2DM and can be a potential biomarker for the prediction of T2DM (32). ALP occurs as a variety of isoenzymes with different roles in the body depending on tissue type. In the liver’s canalicular membrane, it acts as a catalyst for the hydrolysis of organic phosphate esters. ALP is mainly produced by the liver and bone.

### Total protein

Several proteins, including albumin, are produced by the liver. These proteins are important in maintaining osmotic pressure, the transfer of macromolecules, and fighting off infections. Decreased levels of total protein may be an indicator of liver damage. Specifically, serum albumin has been found to have an association with insulin resistance, which primarily induces T2DM (33).

### Bilirubin

Bilirubin is an orange-yellow substance usually found in a conjugated state (34). It is naturally released during the breakdown of heme in erythrocytes by heme oxygenases (HO) (35). Bilirubin is an important molecule in the body due to its antioxidant properties, such as reducing the effect of LDL oxidation and preventing lipid peroxidation (36). T2DM has been shown to cause oxidative stress, which consequently leads to elevated levels of bilirubin (37).

### Liver steatosis

Liver steatosis is an increased level of hepatocellular lipids (HCL). The causes of liver steatosis include elevated levels of free fatty acids and adipocytokines, which consequently leads to high amounts of HCL, as is commonly seen in insulin resistance and T2DM (38). The progression of liver steatosis to nonalcoholic fatty liver disease (NAFLD) can be indicated by mitochondrial and inflammatory malfunction (39).

### Gamma-glutamyl transferase

Gamma-glutamyl transferase (GGT) is an enzyme that plays a role in the metabolism and homeostasis of extracellular reduced glutathione (GSH), an antioxidant (40). GGT is expressed on the luminal surface of ducts and tracts throughout the body, especially in the kidneys. A high level of GGT is considered to be a predictor of T2DM. Patients with T2DM have higher oxidative stress, leading to beta-cell dysfunction in the pancreas followed by insulin resistance and T2DM. To decrease oxidative stress, higher levels of GGT are expressed to increase the antioxidant activity of GSH (41).

In light of the key role of the gut microbiota in the development and progression of liver injury in T2DM, the use of probiotics, prebiotics, and synbiotics has been presented as a potential therapy for the reestablishment of homeostasis (42). Probiotics are defined as live microorganisms commonly found in food that provide health benefits to the host when administered in appropriate quantities, while prebiotics are substrates utilized by these microorganisms to grow and evoke health benefits to the host (43). Mixtures of prebiotics intended to be selectively used by the co-delivered probiotics are referred to as synbiotics (44). All three biotics work towards restoring balance in the gastrointestinal microbiome, especially following dysbiosis, and enhancing the intestinal mucosa and immunity (45). Although several studies have investigated the use of biotics in patients with T2DM, there are no reviews that compare the efficacies of different pro/pre/synbiotic combinations and dosages on hepatic biomarkers among diabetics. This review aims to explore the mechanisms and effectiveness of the use of probiotics, prebiotics, and synbiotics as interventions for the regulation of hepatic biomarkers in patients with T2DM.

## Methods

### Study protocol and search strategy

The protocol for this systematic review was developed using the Preferred Reporting Items for Systematic Reviews (PRISMA). A comprehensive search for published works was undertaken in PubMed, Scopus, Web of Science, Embase, and the Cochrane Library. A gray literature search was performed in ClinicalTrials.org and ProQuest Dissertations and Theses. The primary search was done in June 2020 to examine the effect of probiotics, prebiotics, and synbiotics on hepatic biomarkers in patients with T2DM. A final search was performed in April 2022 to collect any newly published data. A comprehensive breakdown of the search strategy is provided as Supplementary Table 1. Briefly, the search on PubMed consisted of the elements below:

*(“Probiotics”[MeSH Terms] OR “probiotics”[Title/Abstract] OR “probiotic”[Title/Abstract] OR “Prebiotics”[MeSH Terms] OR “prebiotic”[Title/Abstract] OR “prebiotics” [Title/Abstract] OR “Synbiotics”[MeSH Terms] OR “synbiotics”[Title/Abstract] OR “synbiotic”[Title/Abstract] OR “symbiotic”[Title/Abstract] OR “symbiotics” [Title/Abstract] OR “gastrointestinal microbiome”[MeSH Terms] OR “gut microbiome”[Title/Abstract] OR “gut flora”[Title/Abstract]) AND (“diabetes mellitus, type 2”[MeSH Terms] OR “T2D”[Title/Abstract] OR “type 2 diabetes”[Title/Abstract])*.

### Eligibility criteria, screening, and data extraction

Inclusion Criteria: only clinical studies investigating the effect of probiotic, prebiotic, or synbiotic supplementation on hepatic biomarkers in patients diagnosed with T2DM were included. Studies of any duration, involving adults of any age, sex, ethnicity, from any region worldwide, and published at any time, were included.

Exclusion Criteria: studies that included participants diagnosed with other types of diabetes were excluded. Further, we excluded reviews, conference proceedings, abstracts, editorials, animal studies, and other non-clinical forms of literature. Further, records with full texts in non-English languages or those that did not provide details on hepatic biomarkers were omitted. Lastly, we also excluded studies that administered non-bacterial organisms as probiotics or synbiotics.

Studies identified through multi-database searching were imported into Covidence (Veritas Health Innovation, Melbourne, VIC, Australia), which automatically detected and removed duplicates prior to manual screening. At least two independent reviewers systematically screened all remaining studies according to the eligibility criteria. Conflicts were resolved *via* consensus following discussion or by an independent reviewer. All included studies were processed for qualitative analysis and the relevant data from each study was extracted, grouped by themes, and analyzed in the discussion. Extracted data elements from each study included study characteristics, such as first author’s last name, country in which the trial was conducted, study design, trial duration, and investigated biomarker; participant characteristics for both intervention and placebo/control groups, such as mean and standard deviation (SD) of age and baseline body mass index (BMI), total number of participants and ratio of sexes, presence of inclusionary comorbidities; and intervention characteristics such as type, composition, and daily dosage of nutraceutical and control/placebo substance. Changes in liver biomarkers were extracted in the most suitable form provided by the authors and color-coded. This was in the form of intragroup change from baseline to end-of-trial, intergroup mean difference (MD) between intragroup changes, or comparison of both intra-group changes. Classification of nutraceutical type was made after careful examination of nutraceutical formulation.

### Risk of bias assessment

A pre-piloted Excel form, the Cochrane revised risk-of-bias tool version 2 (RoB2), was used for scoring and reporting the risk of bias associated with individual studies (46). Factors that could lead to various risk of bias, including the randomization process, allocation concealment, participant recruitment, deviations from intended intervention, missing outcome data, outcome measurement, and selection of reported results, in addition to overall bias, were rated by independent reviewers. The domains above were scored with low risk, some concerns, or high risk of bias.

## Results

### Search results

**FIGURE 2:**
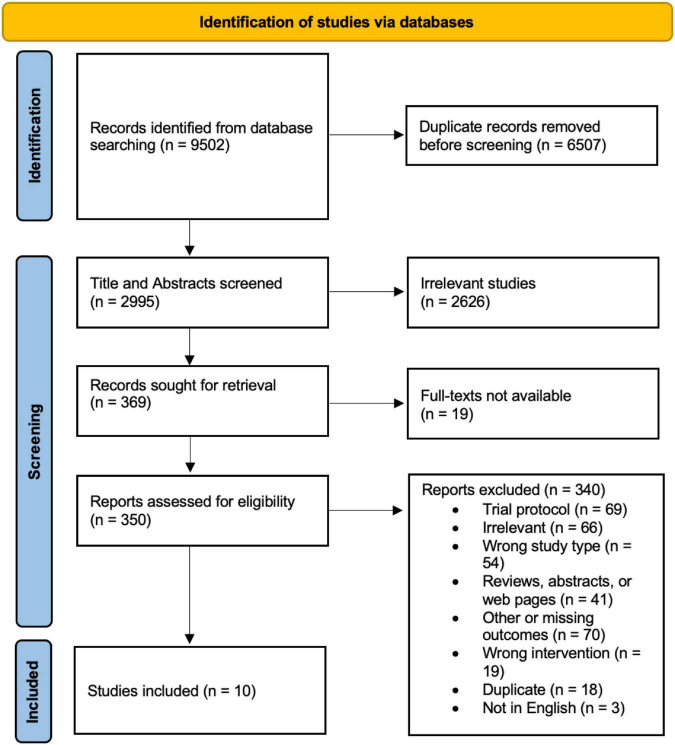
PRISMA study selection flow chart.

The PRISMA study selection protocol flowchart is shown in Figure 2. Briefly, 9,502 records were identified from database searching, of which only 10 were included in the qualitative synthesis and analysis in this review. Of the initial 9,502 records, 6,507 duplicates were removed automatically prior to manual screening. Title and abstract of the remaining 2,995 records were screened, of which 2,626 were found to be irrelevant under the eligibility criteria. Further, 19 full-texts could not be retrieved, leaving 350 reports for full-text screening. Of these, 340 were excluded for various reasons as elucidated in Figure 2, leaving 10 studies for extraction.

### Trial characteristics

In total, 10 studies explored the effects of biotics on hepatic biomarkers, of which 10 studies tested for changes in AST, 10 studies tested ALT, 1 study tested liver steatosis, 6 studies tested ALP, 1 study tested LS, 1 study tested FLI, 1 study tested total protein, 3 studies on bilirubin, and 1 study tested GGT (47–56). The studies involved 816 patients, including 460 in the intervention groups and 356 in the control groups. All subjects were diagnosed with T2DM based on the studies’ individual criteria. Of the studies included, six were from Iran, one from Malaysia, one from Japan, one from Ukraine, and one from Sweden. Median year of publication was 2017 (IQR 2016–2017). The median of the mean age of the intervention groups was 54.1 years (IQR 51.8–59.0), and the median of the mean BMI was 30.6 (IQR 29.8–31.7). The median of intervention periods was 8 weeks. The trials extracted included three studies with prebiotics, three studies with single species probiotics, three studies with multi-species probiotics, and four studies with single species synbiotics. Six genera of bacteria were used in the probiotics and synbiotics, namely *Lactobacillus, Bifidobacterium, Streptococcus, Acetobacter, Lactococcus*, and *Propionibacterium*. Names of the bacterial species, *Lactobacillus sporogenes* (*Bacillus coagulans*), *Lactobacillus acidophilus*, *Limosilactobacillus reuteri*, *Lacticaseibacillus casei*, *Lactococcus lactis*, *Bifidobacterium bifidum*, *Bifidobacterium longum*, *Lacticaseibacillus rhamnosus*, *Lactobacillus delbrueckii* subsp. *bulgaricus*, *Bifidobacterium breve*, and *Streptococcus thermophilus*, were extracted and adjusted based on the most updated nomenclature. The median dosage for probiotics in single and multi-species probiotics and single species synbiotics was 1 × 10^8^ colony forming units per day (CFU/day; IQR: 1 10^8^ to 3.5 10^10^; range: 1 10^7^ to 1 10^11^). The prebiotics used included inulin, inulin and beta carotene, chicory inulin enriched with oligofructose, galacto-oligosaccharides syrup, and powdered cinnamon. The median mass of prebiotics administered both alone and within a synbiotic is 0.5 g/day (IQR: 0.5–9.2; range: 0.04–10).

### Risk of bias and publication bias assessment

Using the Cochrane collaboration RoB2 tool, a risk of bias assessment was performed. All 10 studies had a low risk of bias in the randomization process, participant recruitment, intended intervention, missing outcome data, and outcome measurement and selection (Supplementary Figure 1).

### Effects on alanine transaminase levels

**FIGURE 3:**
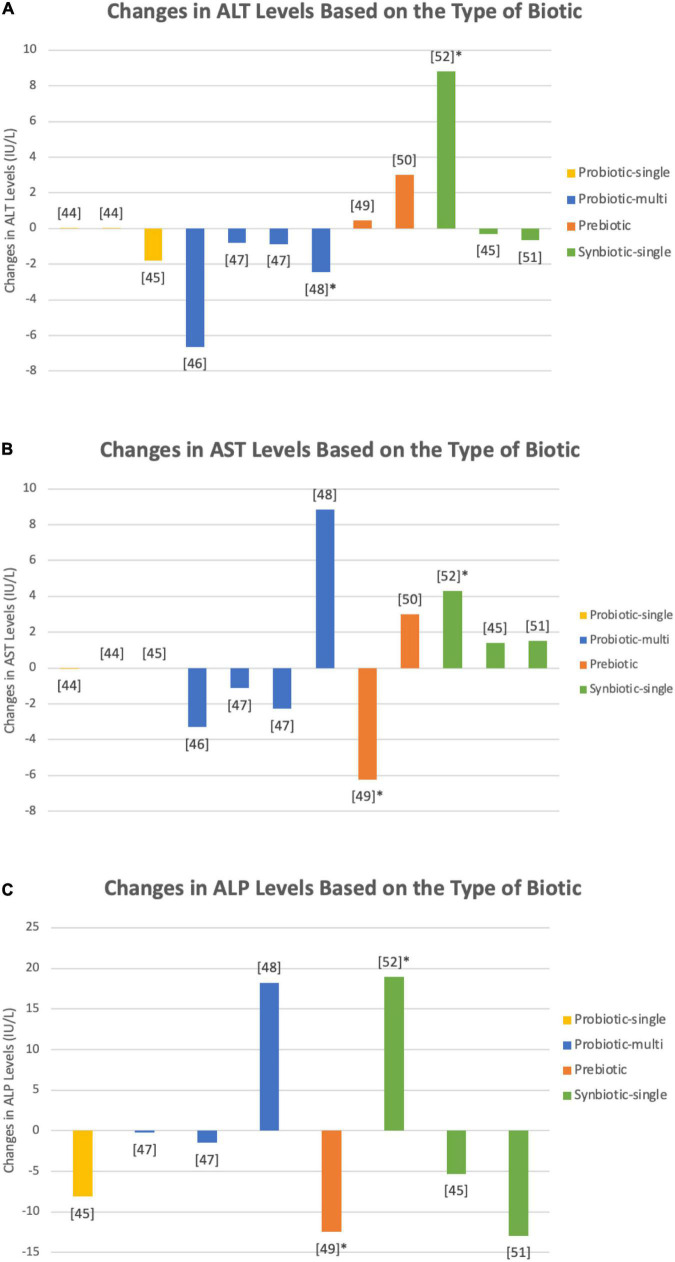
Changes in **(A)** ALP, **(B)** ALT, and **(C)** AST levels upon administration of single species probiotics (yellow), multi-species probiotics (blue), prebiotics (orange), and single species synbiotics (green) in nine studies (47–55). Bars marked with an asterick represent significant changes in the respective biomarker levels. Both single species *L. reuteri* (47) and *B. coagulans* (48) showed insignificant alterations in ALP, ALT, and AST levels. Kobyliak et al. (49) used a multi-species probiotic formulation, comprised of Bifidobacteria and Lactobacilli among others (species not specified), that demostrated a decrease in liver enzymes, however the decrease was insignifcant. Firouzi et al. (50) used a similar multi-species probiotic and tested its effect over two intervention periods, 6 and 12 weeks, both of which yielded no notable alterations in biomarkers. Asemi et al., on the other hand, found a statistically relevant decrease in ALT levels upon administration of a multi-species probiotic containing species of Lactobacilli, Bifidobacteria, and Streptococci (species not specified) (51). Farhangi et al. utilized chicory inulin enriched oligofructose and measured a significant reduction in AST levels (52). Single species synbiotics employed by Asemi et al. (55) combined *B. coagulans* (1 × 10^7^) and inulin (0.04 g), yielding a significant increase in each of ALT, AST, and ALP levels. Synbiotic formulations administered by Asemi et al. (54), containing *B. coagulans* (3 × 10^7^ CFU), inulin, and beta carotene (0.45 g), and Bahmani et al. (48), containing *B. coagulans* (1 × 10^8^ CFU) and inulin (8.4 g), caused no statistically relevant adjustments in hepatic enzyme levels. Mirmiranpour et al. reported significant decreases in ALT and AST levels when subjects were given both prebiotic powdered cinnamon and a synbiotic combination of 1 × 10^8^ CFU of the probiotic *L. acidophilus* with 0.5 g of powdered cinnamon, but no data points were documented in the paper (56).

Table 1 shows a summary of the fourteen interventions in 10 studies that examined the effect of biotics on ALT levels in patients with T2DM (47–56). The interventions consisted of a total of 816 patients, including 460 patients that were given a biotic and 356 given a placebo. As seen in Figure 3A, single species probiotics were administered in four trials, multi-species probiotics in three trials, prebiotics in three trials, and single species synbiotics in four trials. An increase in ALT levels was seen in five trials, while nine trials demonstrated a decrease. In the trials with an increase in ALT levels, two used single species probiotics, two used prebiotics, and one used single species synbiotics. Among the trials that showed a decrease in ALT levels, two trials administered single species probiotics, three administered multi-species probiotics, one administered prebiotic, and three administered single species synbiotics.

**TABLE 1:** Studies investigating changes in hepatic biomarkers following intervention with probiotics, prebiotics, and synbiotics.

Type of nutraceutical	Study design and country	Participant* demographics size/sex (*n*, F/M) age (mean ± SD; years) BMI (mean ± SD; kg/m^2^)		Control/ placebo substance administered	Interventional nutraceutical administered	Control/ placebo and intervention dose × frequency	Intervention duration	Effect on markers	Mean change in markers^Φ^	References
		Control/placebo	Intervention							
Probiotic (single species)	R, DB, C, CT (Iran)	*n* = 27 (12M/15F) 58.2 ± 11.8 BMI NR	*n* = 30 (10M/20F) 59.7 ± 12.2 BMI NR	Capsule containing 0.5 g of rice flour powder	Capsule containing *L. acidophilus* (10^8^ CFU)	1 capsule/day	3 months	↓ AST	Markers NR	Mirmiranpour et al. (56)
								↓ ALT	Markers NR	
Probiotic (single species)	DB, R, PG, PC (Sweden)	T2D and obese patients* n = 15 (11M/4F) 65 ± 5 30.7 ± 4.0	T2D and obese patients*; low dose group *n* = 15 (12M/3F) 66 ± 6 30.6 ± 4.5	Capsule with mildly sweet tasting powder in an aluminum laminate stick pack	Capsule containing low-dose *L. reuteri* DSM 17938 (10^8^ CFU/capsule)	1 capsule/day	12 weeks	↑ Liver steatosis (_§_)	14.0 ± 8.4% fat (I, 12w) vs. 13.9 ± 8.7% fat (I, B) (_§_)	Mobini et al. (47)
								↓ AST (_§_)	0.38 ± 0.11 μkat/L (I, 12w) vs. 0.40 ± 0.14 μkat/L (I, B) (_§_)	
								↑ ALT (_§_)	0.52 ± 0.15 μkat/L (I, 12w) vs. 0.50 ± 0.17 μkat/L (I, B) (_§_)	
			T2D and obese patients*; high dose group *n* = 14 (11M/3F) 64 ± 6 32.3 ± 3.4	Capsule with mildly sweet tasting powder in an aluminum laminate stick pack	Capsule containing high-dose *L. reuteri* DSM 17938 (10^10^ CFU/capsule)	1 capsule/day	12 weeks	↓ Liver steatosis (_§_)	11.3 ± 8.6% fat (I, 12w) vs. 12.0 ± 9.0% fat (I, B) (_§_)	
								⋅ AST (_§_)	0.40 ± 0.12 μkat/L (I, 12w) vs. 0.40 ± 0.12 μkat/L (I, B) (_§_)	
								↑ ALT (_§_)	0.53 ± 0.20 μkat/L (I, 12w) vs. 0.51 ± 0.15 μkat/L (I, B) (_§_)	
Probiotic (single species)	R, DB, PC, CT (Iran)	Control bread (CB) *n* = 27 (5M/22F) 53.4 ± 7.5 30.5 ± 4.1	Probiotic bread *n* = 27 (5M/22F) 52.0 ± 7.2 29.8 ± 5.7	Control bread	Bread containing *L. sporogenes* (1 × 10^8^ CFU/g)	40 × 3 g/day	8 weeks	↓ ALT (_§_)	−1.8 ± 8.3 IU/L vs. +1.4 ± 9.7 IU/L (_§_)	Bahmani et al. (48)
								↑ AST (_§_)	0.01 ± 18.5 IU/L vs. +2.1 ± 14.1 IU/L (_§_)	
								↓ ALP (_§_)	−8.1 ± 31.73 IU/L vs. −5.4 ± 47.3 IU/L (_§_)	
Probiotic (multi-species)	DB, PC, PG, RCT (Ukraine)	Patients with T2D and NAFLD *n* = 24 (NR) 57.38 ± 9.92 32.55 ± 3.62	Patients with T2D and NAFLD *n* = 26 (NR) 53.23 ± 10.09 33.19 ± 4.93	Organoleptically similar formulation as intervention	Symbiter Forte (combination of 250 mg smectite gel) and *Bifidobacterium* (1 × 10^10^ CFU/g), *Lactobacillus* + *Lactococcu*s (6 × 10^10^ CFU/g), *Acetobacters* (1 × 10^6^ CFU/g), and SCFAs producing *Propionibacterium* (3 × 10^10^ CFU/g) genera	10 × 1 g/day	8 weeks	↓ ALT	−6.62 ± 13.07 IU/L or −10.32 ± 32.1%	Kobyliak et al. (49)
								↓ AST	−3.31 ± 6.88 IU/L or −6.20 ± 19.22%	
								↓ LS	−0.254 ± 0.85 kPa (−4.427 ± 12.6%) vs. +0.262 ± 0.77 (+2.38 ± 10.25%)	
								↓ FLI (_§_)	−0.750 ± 1.23 (−1.194 ± 8.43%) vs. +3.769 ± 1.84 (+4.471 ± 12.15%) (_§_)	
Probiotic (multi-species)	R, DB, PC, CT (Iran)	*n* = 30 (sex NS) 52.1 ± 6.9 30.7 ± 4.1	*n* = 28 (sex NS) 49.6 ± 9.9 31.9 ± 6.4	100 mg fructo-oligosaccharide with lactose/capsule	Freeze-dried *L. acidophilus* (2 × 10^9^ CFU), *L. casei* (7 × 10^9^ CFU), *L. rhamnosus* (1.5 × 10^9^ CFU), *L. bulgaricus* (2 × 10^8^ CFU), *B. breve* (2 × 10^10^ CFU), *B. longum* (7 × 10^9^ CFU), *S. thermophilus* (1.5 × 10^9^ CFU), and 100 mg fructo-oligosaccharide with lactose/capsule	1 capsule/day	8 weeks	↑ ALP	+18.25 ± 40.67 mg/dl	Asemi et al. (51)
								↑ ALP (_§_)	+18.25 ± 40.67 mg/dl vs. +4.93 ± 35.91 mg/dl (_§_)	
								↑ AST	+8.86 ± 15.11 mg/dl	
								↑ AST (_§_)	+8.86 ± 15.11 mg/dl vs. +4.11 ± 15.11 mg/dl (_§_)	
								↑ (x)ALT	−2.46 ± 13.10 mg/dl vs. +4.62 ± 10.81 mg/dl	
Probiotic (multi-species)	DB, R, PG, PC (Malaysia)	*n* = 68 (34M/34F) 54.2 ± 8.3 29.3 ± 5.3 *n* = 53 (PP analysis)	*n* = 68 (31M/37F) 52.9 ± 9.2 29.2 ± 5.6 *n* = 47 (PP analysis)	Organoleptically similar sachets without probiotic	Sachets containing viable microbial cell preparation of *L. acidophilus, L. casei, L. lactis, B. bifidum, B. longum*, and *B. infantis* (0.5 × 10^10^ CFU, each) in 250 ml water	2 sachets/day	12 weeks	⋅ Albumin (_§_)	45.48 ± 2.97 g/L (I, 12w) vs. 45.64 ± 3.22 g/L (I, B) (_§_)	Firouzi et al. (50)
								↓ Total protein (_§_)	73.03 ± 5.98 g/L (I, 12w) vs. 74.24 ± 4.93 g/L (I, B) (_§_)	
								↑ Bilirubin (_§_)	10.09 ± 3.70 μmol/L (I, 12w) vs. 9.77 ± 3.50 μmol/L (I, B) (_§_)	
								↓ AST (_§_)	25.71 ± 6.81 U/L (I, 12w) vs. 26.84 ± 77.12 U/L (I, B) (_§_)	
								↓ ALT (_§_)	22.33 ± 10.02 U/L (I, 12w) vs. 23.20 ± 9.65 U/L (I, B) (_§_)	
								↓ ALP (_§_)	67.00 ± 21.77 U/L (I, 12w) vs. 68.49 ± 23.19 U/L (I, B) (_§_)	
Prebiotic	R, DB, C, CT (Iran)	*n* = 27 (12M/15F) 58.2 ± 11.8 BMI NR	*n* = 28 (14M/16F) 58.8 ± 12.8 BMI NR	Capsule containing 0.5 g of rice flour powder	Capsule containing 0.5 g of powdered cinnamon	1 capsule/day	3 months	↓ AST	Markers NR	Mirmiranpour et al. (56)
								↓ ALT	Markers NR	
Prebiotic	DB PC (Iran)	T2D and overweight patients* n = 22 (22F) 48.61 ± 9.16 29.98 ± 4.01	T2D and overweight patients* n = 27 (27F) 48.07 ± 8.70 31.43 ± 3.50	Maltodextrin	Oligofructose-enriched chicory inulin enriched	5 × 2 g/day	2 months	↓ AST	18.02 ± 6.41 U/L (I, 2m) vs. 24.25 ± 12.15 U/L (I, B)	Farhangi et al. (52)
								↑ ALT (_§_)	23.25 ± 12.15 U/L (I, 2m) vs. 22.81 ± 11.04 U/L (I, B)	
								↓ ALP	183.07 ± 48.21 U/L (I, 2m) vs. 195.51 ± 65.20 U/L (I, B); also significant MD vs. control, markers NS	
Prebiotic	R, DB, PC (Japan)	*n* = 25 (17M/8F) 54 ± 12 27.2 ± 4.6	*n* = 27 (21M/6F) 55 ± 11 27.9 ± 3.6	Maltodextrin syrup	Galacto-oligosaccharide syrup	10 g/day	4 weeks	↑ ALT (_§_)	43.0 ± 36.0 IU/L (I, 4w) vs. 40.0 ± 35.0 IU/L (I, B) (_§_)	Gonai et al. (53)
								↑ AST (_§_)	34.0 ± 28.0 IU/L (I, 4w) vs. 31.0 ± 23.0 IU/L (I, B) (_§_)	
Synbiotic (single species)	DB, R, CC, CT (Iran)	*n* = 51 (16M/35F) 52.9 ± 8.1 30.15 ± 5.07	*n* = 51 (16M/35F) 52.9 ± 8.1 29.88 ± 4.77	0.38 g isomalt, 0.36 g sorbitol, and 0.05 g stevia per 1 g	*L. sporogenes* (1 × 10^7^ CFU), 0.1 g inulin, 0.05 g beta-carotene with 0.38 g isomalt, 0.36 g sorbitol, and 0.05 g stevia per 1 g	9 × 3 g/day	6 × 2 weeks	↓ ALP	−12.91 ± 32.65 U/L	Asemi et al. (54)
								↓ ALP (_§_)	−12.91 ± 32.65 U/L vs. −9.40 ± 21.17 U/L (_§_)	
								↓ ALT (_§_)	−0.67 ± 7.42 IU/L vs. +0.67 ± 6.21 IU/L (_§_)	
								↑ AST (_§_)	(+1.52 ± 11.93 IU/L vs. +2.00 ± 8.55 IU/L) (_§_)	
Synbiotic (single species)	R, DB, C, CT (Iran)	*n* = 27 (12M/15F) 58.2 ± 11.8 BMI NR	*n* = 30 (sex NS) 58.4 ± 11.4 30.8 ± 5.9 BMI NR	Capsule containing 0.5 g of rice flour powder	Capsule containing *L. acidophilus* (10^8^ CFU) and 0.5 g of powdered cinnamon	1 capsule/day	3 months	↓ AST	Markers NR	
								↓ ALT	Markers NR	
Synbiotic (single species)	R, DB, CC, CT (Iran)	*n* = 62 (sex NS) 35–70 (age NS) 30.1 ± 5.1	*n* = 62 (sex NS) 35–70 (age NS) 29.7 ± 4.6	0.38 g isomalt, 0.36 g sorbitol, and 0.05 g stevia per 1 g	*L. sporogenes* (1 × 10^7^ CFU), 0.04 g inulin, 0.38 g isomalt, 0.36 g sorbitol, and 0.05 g stevia per 1 g	9 × 3 g/day	6 × 2 weeks	↑ ALP	+18.94 ± 55.50 mg/dl	Asemi et al. (55)
								↑ ALP (_§_)	+18.94 ± 55.50 mg/dl vs. +1.09 ± 59.28 mg/dl (_§_)	
								↑ AST	+4.29 ± 12.17 mg/dl	
								↑ AST (_§_)	+4.29 ± 12.17 mg/dl vs. +4.36 ± 9.53 mg/dl (_§_)	
								↑ ALT	+8.82 ± 22.54 mg/dl	
								↑ ALT (_§_)	+8.82 ± 22.54 mg/dl vs. +3.34 ± 9.39 mg/dl (_§_)	
Synbiotic (single species)	R, DB, C, CT (Iran)	*n* = 27 (5M/22F) 53.4 ± 7.5 30.5 ± 4.1	*n* = 27 (5M/22F) 51.3 ± 10.4 30.8 ± 5.9	Control bread	Bread containing viable and heat-resistant *L. sporogenes* (1 × 10^8^ CFU) and 0.07 g inulin per gram	40 × 3 g/day	8 weeks	↓ ALT (_§_)	−0.3 ± 10.9 IU/L vs. +1.4 ± 9.7 IU/L (_§_)	Kobyliak et al. (49)
								↓ GGT (§)	−1.36 ± 44.22 IU/L vs. −0.76 ± 25.92 IU/L (§)	
								↑ AST (_§_)	+1.4 ± 14.3 IU/L vs. +2.1 ± 14.1 IU/L (_§_)	
								↓ ALP (_§_)	−5.3 ± 60.0 IU/L vs. −5.4 ± 47.3 IU/L (_§_)	

*All participants are T2D-diagnosed patients, unless otherwise stated. ^Φ^ Order of markers compared = those of intervention (I) group first, control (B) or baseline (B) second. ^§^ Non-significant result. T2D, type 2 diabetes; NS, not specified; NR, not reported; Sp., species; SB, single-blinded; DB, double-blinded; TB, triple-blinded; R, randomized; RCT, randomized controlled trial; CC, crossover controlled; PC, placebo-controlled; PG, parallel group; CT, clinical trial; UCS, uncontrolled study; ALP, alkaline phosphatase; ALT, alanine aminotransferase; AST, aspartate aminotransferase; LS, liver stiffness; FLI, fatty liver index; LDH, lactate dehydrogenase; γGGT, gamma-glutamyl transferase.

### Effects on aspartate transaminase levels

There were 14 interventions in 10 studies on the effects of biotics on AST levels, as summarized in Table 1 (47–56). The trials included 816 patients, of which 460 patients were given a biotic and 356 patients were given a placebo. Depicted in Figure 3B, single species probiotics were used in four trials, multi-species probiotics were used in three trials, prebiotics were used in three trials, and single species synbiotics were used in four trials. Out of the 14 interventions, 6 reported an increase in AST levels upon administration of pro/pre/synbiotics, 7 reported a decrease, and 1 reported no change. Of the interventions that demonstrated an increase in AST levels, one used single species probiotics, one used multi-species probiotics, one used prebiotic, and three used single species synbiotics. Of the interventions with a decrease in AST levels, two offered single species probiotics, two multi-species probiotics, two offered prebiotics, and one offered single species synbiotics. No change in AST levels was observed when patients were given a single species probiotic.

### Effects on alkaline phosphatase levels

As summarized in Table 1, seven interventions in six studies examined the effect of different biotics on ALP levels (48, 50–52, 54, 55). A total of 555 patients with T2DM were employed, 290 participants in the intervention groups and 265 participants in the control group. Figure 3C portrays one intervention that examined the effects of prebiotics, one that examined single species probiotics, two that examined multi-species probiotics, and three that examined single species synbiotics. Overall, two of the seven interventions reported an increase in ALP levels upon administration of biotics, and five reported a decrease. Of the studies that observed an increase in the marker’s levels, one used multi-species probiotics, and one used a single species synbiotic. Of the interventions that found a decrease, one utilized prebiotic, one utilized single species probiotics, one utilized multi-species probiotics, and two utilized single species synbiotics.

### Effects on liver steatosis

Only two interventions in one study reported the effects of biotics on liver steatosis (Table 1) (47). The study employed 44 participants with T2DM, 29 of which were in the intervention group and 15 were in the control group. Both trials used the same type of single species probiotic, with an increase in HCL in one trial that used a dose of 10^10^ CFU (*n* = 14) and a decrease in HCL in the second trial that used a lower dose of 10^8^ CFU (*n* = 15).

### Effects on liver stiffness and fatty liver index

One intervention from one study tested the effects of multi-species probiotics on both LS and fatty liver index (FLI) (Table 1) (49). The intervention consisted of 50 patients with T2DM, 26 of which were given the probiotics and 24 were given a placebo. Both LS and FLI decreased after 8 weeks of taking the probiotics.

### Effects on total protein levels

Table 1 shows one intervention from one study that looked into the changes in total protein levels in T2DM patients after taking multi-species probiotics (50). A total of 136 patients were employed in these trials, 68 of which were administered multi-species probiotics and 68 took a placebo. The trial yielded a decrease in total protein levels after a 6- and 12-week intervention period.

### Effects on bilirubin levels

There were three interventions in three studies that demonstrated the effects of biotics on bilirubin levels (Table 1) (50, 51, 55). The interventions collectively consisted of 318 participants, 158 of whom were in the intervention group and 160 in the control group. Two trials used multi-species probiotics, while the third trial tested single species synbiotics. One of the interventions reported an increase in bilirubin levels using multi-species probiotics. The other two reported a decrease; one trial also used multi-species probiotics, while the other used single species synbiotics.

### Effects on gamma-glutamyl transferase levels

Only one intervention from one study reported the effects of biotics, specifically multi-species probiotics on the levels of GGT (Table 1) (49). A total of 50 participants were employed, 26 of which were given the multi-species probiotic and 24 of which were given a placebo. GGT levels decreased after administration of multi-species probiotics across an 8-week interventional period.

## Discussion

There is a growing interest in the relationship between the gut microbiome, its dysbiosis and the pathophysiology of various diseases, including T2DM. In turn, there has been a recent increase in the number of studies investigating the effect of pro/pre/synbiotics as microbiome-modulating agents to utilize their potential therapeutic potential against the metabolic imbalances observed in T2DM patients (57). Thus, there is a significant need to qualitatively summarize, analyze, and provide future directives for investigation in this field. To our knowledge, this is the most comprehensive and detailed systematic review investigating the effect of pro/pre/synbiotics on hepatic biomarkers in clinical trial participants with T2DM.

### Current and future directives for probiotics

Single-species probiotic formulations, especially including *Lactobacillus* species, have been consistently investigated for their effect on various metabolic diseases. Bahmani et al. performed a double-blind, placebo-controlled trial on 81 diabetic patients to examine the effects of both probiotic and synbiotic bread on liver enzymes, among other biomarkers (48). The consumption of probiotic bread containing *L. sporogenes* (1 10^8^ CFU), currently referred to as *B. coagulans*, caused an insignificant decrease in ALP (*p* = 0.97) and ALT (*p* = 0.48) levels, and an insignificant increase in AST levels (*p* = 0.88). Although these results may be limited by the short intervention period (8 weeks), some scientists have questioned whether these spores are excreted intact from patients and therefore have no probiotic effect (58). While the vegetative state of this bacterium facilitates transportation and quality of the probiotic, *B. coagulans* may not survive long in the harsh conditions of the gut. Hibernating in its dormant form, the probiotic was shown to have weak tolerance, if not sensitivity, to bile, delaying its proliferation by up to 60 min (59). Additionally, one study suggested that *B. coagulans* has weak adhesion to the intestinal epithelium of piglets, being lost a week after administration (59). Such characteristics may explain the insignificant effects on the above-mentioned liver enzymes. Nonetheless, Bahmani et al. did find a significant increase in nitrous oxide (NO) (*p* < 0.001) and decrease in malondialdehyde upon administration of *B. coagulans* (*p* = 0.001), which may indicate a potential application in liver regeneration and reduced lipid oxidation (60). Mirmiranpour et al. reported a significant decrease in ALT and AST levels upon daily administration of 1 10^8^ CFU of the probiotic *L. acidophilus* for 3 months (56). Although the exact mechanism for this decrease is not well understood, several studies highlighted the effect of *L. acidophilus* on the inhibition of pro-inflammatory cytokines (56, 61). Lv et al. fed rats *L. acidophilus* (3 10^9^ CFU) for 7 days and observed an initial alleviation of histological hepatic injury, in addition to a suppression of macrophage inflammatory cytokines, leading to a reduction in serum ALT, AST, ALP, and bile acids (62). *L. acidophilus* also plays a role in the minimization of gut dysbiosis. On the other hand, Mobini et al. reported various changes over the course of 12 weeks in AST, ALT, and liver steatosis levels upon administration of different doses of the probiotic *L. reuteri* (1 10^8^ CFU and 1 10^10^ CFU), although the changes were not significant (47). The insignificant results could be due to some of the study’s limitations, including the subjects’ consumption of metformin, an antidiabetic drug, which is known to affect gut microbiota composition, as well as the relatively small cohort size (47).

Based on the studies analyzed above, the use of single species probiotic *L. acidophilus* (1 10^8^ CFU) demonstrated a significant improvement in hepatic function compared to other species within the same genus. In fact, it has been demonstrated that *Lactobacillus* has an effect on various diseases, especially liver diseases (61, 63). The use of different species of *L. acidophilus* on mice showed that it could improve intestinal barrier function, restore the composition of the gut microbiota, increase SCFA levels to that of the control group, suppress inflammatory responses in the liver and regulate glucose and lipid metabolism in the liver, hence improving T2D (64). One of the potential mechanisms that might explain the beneficial effect of *L. acidophilus* is that this organism can reshape the composition of the gut microbiota, leading to an increase in butyric acid that targets the liver (64, 65).

Multi-species probiotics have also been investigated for their effects in the physiological modulation of the intestinal microbiota as a result of the diversity in the administered species. One study revealed insignificant changes in ALT, AST, ALP, bilirubin, and total protein levels (*p* = 0.199, *p* = 0.441, *p* = 0.209, *p* = 0.739, and *p* = 0.190, respectively) when T2DM participants were administered 6 10^10^ CFU of a multi-species probiotic containing *L. acidophilus*, *L. casei*, *L. lactis*, *B. bifidum*, *B. longum*, and *Bifidobacterium infantis* over 12 weeks (50). As opposed to the anti-inflammatory and antioxidant properties observed in the administration of single species *L. acidophilus*, when given in addition to several other probiotic species, *L. acidophilus* may have an antagonistic effect (66). A study conducted by Kwoji et al. highlighted the complexity of the interactions between various multi-species probiotics and their effects on human health, inhibition of pathogens, and treatment of disease (66). While *L. acidophilus* alone yields a significant reduction in several hepatic biomarkers due to decreased cellular injury and apoptosis, consequently leading to mitigation of the release of these intracellular enzymes (67), its administration with other species may not produce such effects and may require further investigation.

Another study conducted by Asemi et al. examined the effects of daily administration of multi-species probiotics containing a combined dose of 3.72 10^10^ CFU of *L. acidophilus*, *L. casei*, *L. rhamnosus*, *L. delbrueckii* subsp. *bulgaricus*, *B. longum*, *B. breve*, and *S. thermophilus* over an 8-week intervention period (51). Asemi et al.’s trials yielded insignificant changes in ALP, AST, and bilirubin levels (*p* = 0.19, *p* = 0.23, and *p* = 0.91, respectively) but, as seen in Figure 3A, a significant decrease in ALT levels (*p* = 0.02). Multi-species probiotics have complex interactions that, while unfavorable at times, may have combined benefits for overall human health (66), detectable as improvements in liver biomarker levels, namely ALT and AST. Although Asemi et al. portrayed an increase in the levels of AST as well as uncertainty regarding the changes of other liver biomarkers, these changes were insignificant. Finally, Kobyliak et al. provided 1 10^11^ CFU of multi-species probiotic daily containing Bifidobacteria, Lactobacilli, Lactococci, Acetobacters, and SCFA-producing Propionibacteria (species not specified) (49). Although the study reported decreases in ALT, AST, FLI, LS, and GGT after an 8-week intervention period, none of these changes were significant (*p* = 0.991, *p* = 0.420, *p* = 0.521, *p* = 0.401, and *p* = 0.088, respectively). While it is true that many probiotics may have synergistic effects when administered with one another, some probiotics alone and in conjunction with others may prove to have antagonistic effects on the host’s microbiota (68). Since the authors never specified the specific species of bacteria used in the intervention, certain harmful *Lactobacillus* species may have been used in conjunction with possibly beneficial species to yield inconsistent findings, hence explaining the insignificant reduction of the previously listed markers (68). Furthermore, the dosage could have also contributed to the insignificant results as the combined dose is marginally higher than the recommended ranges provided by Islam et al. which may explain a potential reduction in these liver biomarkers (69).

While it is unclear that specific combinations of probiotics yielded beneficial hepatic effects, the species *L. acidophilus*, *L. casei*, and B. *longum* were consistently used throughout all three studies and may be associated with an improvement of hepatic function (49–51). However, specific doses and intervention durations may need adjustment to clarify the effects of these variables and optimize the effects of the multi-species intervention. More in-depth investigations may be needed to assess the intricate mechanisms governing the complex interactions between multi-species probiotics to understand the end results on various liver biomarkers.

### Current and future directives for prebiotics

Prebiotics have been investigated in several studies as low-risk, low-cost supplements to conventional T2DM treatments (Figures 3A–C). Farhangi et al. studied the effects of chicory inulin enriched with oligofructose on liver function tests, as well as glucose and calcium homeostasis, in female T2DM patients (52). Upon the administration of 10 g of the prebiotic for 2 months, a significant decrease was recorded in both ALP and AST levels compared to baseline (*p* = 0.05 and *p* < 0.001, respectively), but there was an insignificant increase in ALT levels (*p* = 0.39). Inulin and oligofructose are functional foods commonly found in plants, and they have been theorized to aid in important physiological processes, including modulating the gut microbiota’s composition (70). The underlying mechanism of action of inulin oligofructose is the selective “fertilization” of SCFA-producing bacteria, such as Bifidobacteria, Lactobacilli, and Bacteroides (52). Chicory inulin is broken down by bacterial groups *via* β-fructofuranosidase, generating fermented byproducts such as acetate, lactate, and propionate (71–73). These substances play key roles in maintaining homeostasis, reducing inflammation, and alleviating insulitis (74). Gut microbiota changes, reduction in endotoxemia and insulin resistance, and improvement in glycemic control were also observed by Ho et al. (75), who provided oligofructose enriched inulin to children with T1D. These findings present chicory inulin enriched with oligofructose as a potential complement to current biomedical treatments. Galacto-oligosaccharide, another type of prebiotic, was given to T2D patients by Gonai et al., but no notable changes were recorded in hepatic biomarkers (53). Gonai et al. also investigated and compared the gut microbiota’s composition and metabolites in T2DM patients versus a control group, as well as the effect of galacto-oligosaccharide ingestion on lipid blood profile and glucose indices in T2DM patients (53). Analyses revealed a significantly lower abundance of Bifidobacteriaceae in T2DM patients before treatment, as well as Actinobacteria, Lachnospiraceae, and Firmicutes (*p* < 0.05), and these results have been supported by other studies (76). On the other hand, Lachnospiraceae were found to be positively correlated with AST and ALT levels, which may be indicative of high lipid metabolism corresponding to a metabolic disturbance (77, 78). After taking the galacto-oligosaccharides, the levels of the above-mentioned bacteria in T2DM patients changed significantly (*p* < 0.05), promoting eubiosis and helping with the regulation of liver function. The assessment showed no significant change in AST and ALT levels upon consumption of the prebiotic; however, this may be due to comparatively shorter intervention durations and sample sizes. Significant decreases in ALT and AST, after a 3 month follow-up, were also reported by Mirmiranpour et al. when a daily dose of 0.5 g of powdered cinnamon was given to subjects (56). Cinnamon has been shown to have therapeutic effects when consumed in adequate amounts. A study by Shekarchizadeh-Esfahani et al. concluded that administering cinnamon at daily dosages of <1,500 mg for at least 12 weeks significantly reduced ALT levels (*p* = 0.002) (79). Although cinnamon’s effect on liver function remains unclear, several studies reported cinnamon’s interaction with peroxisome proliferator-activated receptor (PPAR) which ultimately improves insulin resistance, down-regulates pro-inflammatory cytokine levels, and decreases serum AST levels (79, 80). Longe et al. also looked at the hepatoprotective properties of cinnamon on alloxan induced diabetic rats (81). The results were promising, yielding decreased ALT, AST, and ALP levels, as well as other improvements in glycemic and lipid profiles.

Overall, observations of the effects of prebiotics on hepatic functions has yielded remarkable results. The use of prebiotics, such as cinnamon and chicory inulin enriched with oligofructose, neutralized symptoms of liver damage, consequently decreasing liver enzyme levels, and also helped restore gut microbiota eubiosis.

### Current and future directives for synbiotics

Synbiotics have been investigated for their synergistic potential stemming from combination of pro/prebiotics. A study by Asemi et al. reported insignificant changes in the liver enzymes AST, ALP, and ALT upon intake of beta-carotene fortified single species synbiotic comprising *B. coagulans* (3 10^7^ CFU), inulin, and beta carotene (0.45 g) (54). Bahmani et al. also did not find any significant changes in liver enzymes when a synbiotic formulation of *B. coagulans* (1 10^8^ CFU) and inulin (8.4 g) was used (48). Although Farhangi et al. demonstrated a significant decrease in liver enzyme levels upon administration of chicory inulin enriched with oligofructose alone in diabetic patients, combining the prebiotic with the probiotic *B. coagulans* and beta carotene in the synbiotic produced no notable changes in hepatic biomarkers (52). This lack of significant reduction of serum liver enzymes may be explained by the short duration periods as well as the dose of the probiotic and prebiotics. There are currently limited data on the potential benefits of *B. coagulans* on the gut microbiota and liver function. Interestingly, though, when Asemi et al. used a lower daily dose of both the *B. coagulans* (1 10^7^) and inulin (0.04 g) on T2DM patients, there was a significant increase in serum concentrations of ALP (*p* = 0.009), ALT (*p* = 0.003), and AST (*p* = 0.007), with a significant drop in bilirubin levels (*p* = 0.007) (55). Further analyses may be needed to determine the potential use and mechanism of this synbiotic combination in T2DM patients. Be that as it may, significant decreases in ALT and AST levels were reported by Mirmiranpour et al. following a trial that combined daily administration of 1 10^8^ CFU of the probiotic *L. acidophilus* with 0.5 g of powdered cinnamon over 3 months (56). The results were remarkable as they did not outperform *L. acidophilus* and cinnamon alone. Despite several other studies indicating the benefit of synbiotics in improving antioxidant and anti-inflammatory indices, these studies did not obtain their results from diabetic patients (82–84). While cinnamon and *L. acidophilus* may work additively or synergistically as potential therapeutic compliments in patients with T2DM, their interactions may be altered based on the patients’ comorbidities and atypical hepatic functions. A meta-analysis discussing the effects of probiotics and synbiotics on liver and renal biomarkers in T2DM patients obtained results running mostly in parallel to this paper, with some exceptions (85): even though Abdollahi et al. concluded with insignificant changes in liver biomarkers overall, their results were based on a smaller sample size with limited focus on the isolated effects of the nutraceuticals studied (85).

While the use of synbiotics was not effective in improving hepatic function, exceptions applied to the use of cinnamon and *L. acidophilus*, although their use together yielded similar results to their use as isolated components. Further studies are needed to investigate the mechanism of action of symbiotic components to understand their potential conflicting effects when administered together.

### Limitations and strengths

There are a few limitations to this study. First, due to the great heterogeneity between the types of nutraceuticals, their composition and dosage, the duration of intervention, and the diversity of the trial participant characteristics, it was difficult to ascertain the optimum combination of the above factors that provided the greatest effect on hepatic biomarkers. Second, our search strategy for prebiotics captured only those trials that explicitly self-identified the use of prebiotics; this has a potential to omit other sources (86, 87). Moreover, we did not identify adverse events following administration for nutraceuticals, although prior studies have shown minimal complications. Finally, the sample sizes of most trials are small. Future high-quality studies should have larger sample sizes, longer durations, and more regionally diverse participants. However, this study has multiple strengths. It is one of the most comprehensive reviews of the effect of pro/pre/synbiotics on liver profiles in T2D patients, whereas other reviews largely focus on probiotics only or in addition to synbiotics. Bias in study selection was minimized *via* independent screening and extraction of studies, and RoB assessment revealed low risk of bias for most parameters within the studies used.

## Conclusion and future prospects

This systematic review examined the potential medicinal uses of probiotics, prebiotics, and synbiotics in improving liver function in patients with T2DM. Present evidence reveals that insulin resistance, oxidative stress, and gut dysbiosis contribute to fluctuations in hepatic biomarkers. Supplementation of some biotic formulations, such as prebiotic chicory inulin enriched with oligofructose and multi-species probiotics, demonstrated statistically significant improvements in liver function, specifically in the levels of liver enzymes. However, several studies showed no significant changes or significant increases in these biomarkers upon administration of specific species and types of probiotics and prebiotics. Such contradictory data may be due to differences in doses, intervention periods, or species of probiotics used. Nevertheless, more research needs to be done to better assess the best dose-response relationships for the biotics mentioned in this paper.

## Data availability statement

The original contributions presented in the study are included in the article/Supplementary material, further inquiries can be directed to the corresponding author.

## Author contributions

AC designed the study, critically supervised the project, revised and reviewed the manuscript, and initially screened studies. YA-N and MA analyzed the data, updated the search, wrote the majority of the manuscript, generated the figures, and edited the manuscript. PP initially screened and extracted studies, generated the tables, wrote parts of the manuscript, and reviewed all of it. All authors have read and agreed to the published version of the manuscript.
