# Supplementary material for: Can probiotic, prebiotic, and synbiotic supplementation modulate the gut-liver axis in type 2 diabetes? A narrative and systematic review of clinical trials
Source: Front Nutr. 2022 Dec 1;9:1052619. doi: 10.3389/fnut.2022.1052619 (PMC9751375; doi:10.3389/fnut.2022.1052619)

Supplementary Table ST1. Detailed Search Strategy in Each Database, last updated 10 April 2022**.**

| Database | Search Strategy | |
| --- | --- | --- |
| PubMed | ("Probiotics"[MeSH Terms] OR "probiotics"[Title/Abstract] OR "probiotic"[Title/Abstract] OR "Prebiotics"[MeSH Terms] OR "prebiotic"[Title/Abstract] OR "prebiotics"[Title/Abstract] OR "Synbiotics"[MeSH Terms] OR "synbiotics"[Title/Abstract] OR "synbiotic"[Title/Abstract] OR "symbiotic"[Title/Abstract] OR "symbiotics"[Title/Abstract] OR "gastrointestinal microbiome"[MeSH Terms] OR "gut microbiome"[Title/Abstract] OR "gut flora"[Title/Abstract]) AND ("diabetes mellitus, type 2"[MeSH Terms] OR "T2D"[Title/Abstract] OR "type 2 diabetes"[Title/Abstract]). Limit to clinical and animal studies. | |
| Scopus | (INDEXTERMS ("clinical trials" OR "clinical trials as a topic" OR "randomized controlled trial" OR "Randomized Controlled Trials as Topic" OR "controlled clinical trial" OR "Controlled Clinical Trials" OR "random allocation" OR "Double-Blind Method" OR "Single-Blind Method" OR "Cross-Over Studies" OR "Placebos" OR "multicenter study" OR "double blind procedure" OR "single blind procedure" OR "crossover procedure" OR "clinical trial" OR "controlled study" OR "randomization" OR "placebo")) OR (TITLE-ABS-KEY (("clinical trials" OR "clinical trials as a topic" OR "randomized controlled trial" OR "Randomized Controlled Trials as Topic" OR "controlled clinical trial" OR "Controlled Clinical Trials as Topic"  OR  "random allocation"  OR  "randomly allocated"  OR  "allocated randomly"  OR  "Double-Blind Method"  OR  "Single-Blind Method"  OR  "Cross-Over Studies"  OR  "Placebos"  OR  "cross-over trial"  OR  "single blind"  OR  "double blind"  OR  "factorial design"  OR  "factorial trial" ) ) )  OR  ( TITLE-ABS ( clinical  AND trial* OR  rct*  OR  random*  OR  blind* ) ) AND  ( ( ( TITLE-ABS-KEY ( probiotics  OR  probiotic  OR  prebiotic  OR  prebiotics ) )  OR  ( TITLE-ABS-KEY ( synbiotics  OR  synbiotic  OR  symbiotic  OR  symbiotics ) )  OR  TITLE-ABS-KEY ( "gastrointestinal microbiome" )  OR  TITLE-ABS-KEY ( "gut microbiome" )  OR  TITLE-ABS-KEY ( "gut flora" ) AND  ( TITLE-ABS-KEY ( diabetes  AND mellitus  AND type  2 )  OR  TITLE-ABS-KEY ( t2d )  OR  TITLE-ABS-KEY ( type  2  diabetes ) ) ) ) | |
| Web of Science | TOPIC ((probiotic* OR prebiotic* OR sy*biotic OR symbiotic* OR synbiotic*) OR ("gastrointestinal microbiome" OR "gut microbiome" OR "gut flora")) AND TOPIC ("diabetes mellitus type 2" OR t2d OR "type 2 diabetes"). Limit to: Clinical Trials. | |
| Embase | (probiotic*.mp. [mp=title, abstract, heading word, drug trade name, original title, device manufacturer, drug manufacturer, device trade name, keyword, floating subheading word, candidate term word] OR prebiotic*.mp. [mp=title, abstract, heading word, drug trade name, original title, device manufacturer, drug manufacturer, device trade name, keyword, floating subheading word, candidate term word] OR sy*biotics.mp. [mp=title, abstract, heading word, drug trade name, original title, device manufacturer, drug manufacturer, device trade name, keyword, floating subheading word, candidate term word] OR sy*biotic.mp. [mp=title, abstract, heading word, drug trade name, original title, device manufacturer, drug manufacturer, device trade name, keyword, floating subheading word, candidate term word] OR gut flora.mp. [mp=title, abstract, heading word, drug trade name, original title, device manufacturer, drug manufacturer, device trade name, keyword, floating subheading word, candidate term word] OR gastrointestinal microbiome.mp. [mp=title, abstract, heading word, drug trade name, original title, device manufacturer, drug manufacturer, device trade name, keyword, floating subheading word, candidate term word] OR gut microbiome.mp. [mp=title, abstract, heading word, drug trade name, original title, device manufacturer, drug manufacturer, device trade name, keyword, floating subheading word, candidate term word]) AND (diabetes type 2.mp. [mp=title, abstract, heading word, drug trade name, original title, device manufacturer, drug manufacturer, device trade name, keyword, floating subheading word, candidate term word] OR t2d.mp. [mp=title, abstract, heading word, drug trade name, original title, device manufacturer, drug manufacturer, device trade name, keyword, floating subheading word, candidate term word] OR type 2 diabetes.mp. [mp=title, abstract, heading word, drug trade name, original title, device manufacturer, drug manufacturer, device trade name, keyword, floating subheading word, candidate term word] OR diabetes mellitus.mp. [mp=title, abstract, heading word, drug trade name, original title, device manufacturer, drug manufacturer, device trade name, keyword, floating subheading word, candidate term word]. Limit to (clinical trial or randomized controlled trial or controlled clinical trial or multicenter study or phase 1 clinical trial or phase 2 clinical trial or phase 3 clinical trial or phase 4 clinical trial) | |
| Clinical Trials | (“Diabetes Mellitus, Type 2” OR “Type 2 diabetes” OR Diabetes) AND (prebiotic OR probiotic OR symbiotic OR synbiotic OR "gastrointestinal microbiome" OR “Gut Microbiota”). Filtered by Completed Studies | |
| ProQuest Dissertations and Theses | (Probiotic* OR Prebiotic* OR Synbiotic* OR Symbiotic* OR "gastrointestinal microbiome" OR "Gut Flora" OR "gut microbiome") AND ab(diabetes AND ("type 2" OR t2d OR "Type II")) | |
| Cochrane | 1      MeSH descriptor: [Probiotics] | 12   "gut flora" |
|  | 2      probiotic | 13   "gastrointestinal microbiome" |
|  | 3      prebiotic | 14   "gut microbiome" |
|  | 4      MeSH descriptor: [Prebiotics] | 15   "diabetes mellitus" |
|  | 5      symbiotic | 16   MeSH descriptor: [Diabetes Mellitus] |
|  | 6      synbiotic | 17   t2d |
|  | 7      MeSH descriptor: [Synbiotics] | 18   "type 2" AND diabetes |
|  | 8      probiotics | 19   1 or 2 or 3 or 4 or 5 or 6 or 7 or 8 or 9 or 10 or 11 or 12 or 13 or 14 |
|  | 9      prebiotics | 20   15 or 16 or 17 or 18 |
|  | 10   sy*biotics | 21   19 and 20 |
|  | 11   MeSH descriptor: [Gastrointestinal Microbiome] | 22   Limit to: Controlled trials |

Supplementary Figure SF1. Risk of Bias Assessment


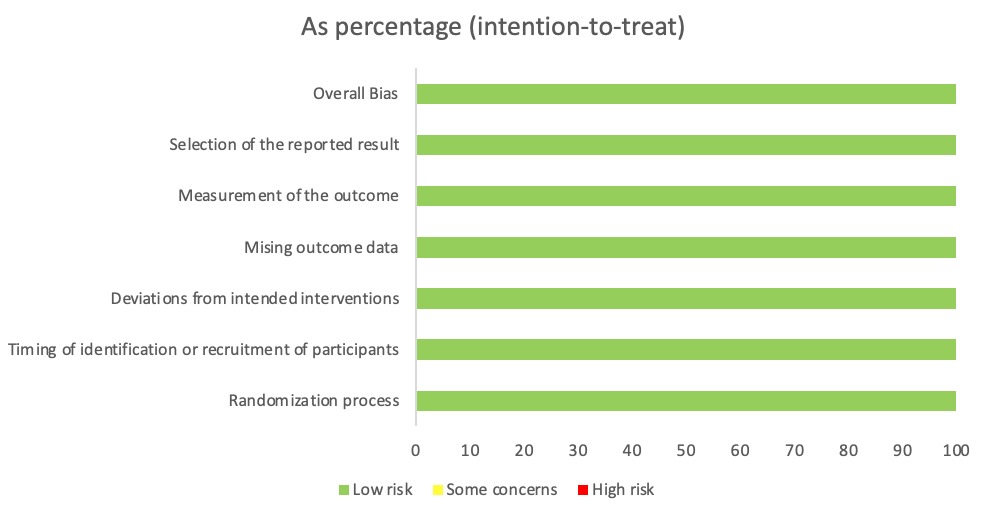

Supplement: Supplementary file 1 [file Data_Sheet_1.docx]
